# Ascorbic and salicylic acid regulate antioxidant defense and drought stress responses in maize

**DOI:** 10.1080/15592324.2026.2664295

**Published:** 2026-05-08

**Authors:** Areeba Abdul Khaliq, Muhammad Shahbaz Naeem, Kaleem ul Din, Muhammad Ahmad, Usman Zulfiqar, Md Opu Sarker, Umair Ashraf, Sajad Ali, Gamal Awad El-Shaboury, Abdulrahman Alasmari, Mohd Asif Shah

**Affiliations:** a Department of Botany, University of Agriculture, Faisalabad, Pakistan; b Department of Agronomy, University of Agriculture, Faisalabad, Pakistan; c West Central Research, Extension & Education Center, University of Nebraska-Lincoln, North Platte, NE, United States; d Department of Agronomy & Horticulture, University of Nebraska-Lincoln, Lincoln, NE, United States; e Department of Agronomy, Faculty of Agriculture and Environment, The Islamia University of Bahawalpur, Bahawalpur, Pakistan; f Department of Biology, Nakhchivan State University, Nakhchivan, Azerbaijan; g Department of Botany, Division of Science and Technology, University of Education, Lahore, Punjab, Pakistan; h Upper Peninsula Research and Extension Center, Michigan State University, Chatham, MI, United States; i Department of Biological Sciences, College of Science, King Faisal University, Al-Ahsa, Saudi Arabia; j Department of Biology, College of Science, King Khalid University, Abha, Saudi Arabia; k Department of Biology, Faculty of Science, University of Tabuk, Tabuk, Saudi Arabia; l Biodiversity Genomics Unit, Faculty of Science, University of Tabuk, Tabuk, Saudi Arabia; m Kardan University, Parwane Du, Kabul, Afghanistan; n Division of Research and Development, Lovely Professional University, Phagwara, Punjab, India; o University Centre for Research & Development, Chandigarh University, Gharuan, Mohali, Punjab, India

**Keywords:** Antioxidant defense, oxidative stress, stomatal regulation, stress mitigation

## Abstract

Climate change led to water scarcity becoming the greatest threat to the growth and yield of maize. Despite the application of growth promoters, the synergistic effects of ascorbic and salicylic acid application remain unexplored, regarding the biochemical mechanisms in the maize plant under drought stress conditions. This study aimed to investigate the impact of foliar applications of ascorbic and salicylic acid on inducing drought stress tolerance in maize. The treatments were comprised of drought (WW = well-watered at 85% FC, DS = drought stress at 40% FC), foliar application (NH = no-spray, DWS = distilled water spray, AA2 = ascorbic acid (200 mg/L), SA2 = salicylic acid (200 mg/L), and AA + SA = combined application (100 mg/L + 100 mg/L). Results showed that drought stress decreased morphological attributes such as shoot length (16%) and leaf area index (11%), photosynthetic pigments like total chlorophyll (15%), and carotenoid levels (19%). However, the combined foliar application of AA + SA significantly increased the above-mentioned morphological attributes (44% and 37%) and photosynthetic pigments (47% and 43%) among the other treatments. Drought stress increased the overproduction of stress indicators such as malondialdehyde (MDA) by 26% and hydrogen peroxide (H_2_O_2_) by 23%. However, AA + SA application reduced the overproduction of these stress indicators by 36% and 32%, through improving the activity of enzymatic antioxidants (superoxide dismutase by 45% and catalase by 38%) and the accumulation of osmolytes (proline by 46% and total soluble proteins by 20%). Overall, foliar treatment with AA2 and SA2 is a long-term approach for improving maize defense mechanisms, resulting in increased plant health and production under drought-stress environments.

## Introduction

One of the largest and most significant crops in worldwide is maize. It is cultivated in most parts of Pakistan across a variety of soil types and climates.^1^ Well-drained, fertile loamy soils are ideal for its growth. Input management, particularly in relation to irrigation water, necessitates the careful application of contemporary technology to optimize output and yield a favorable return.^2^ Drought stress has a significant impact on maize production during the most crucial growth period, which spans two weeks prior to and two to three weeks following silking. For optimal growth and output, irrigation should be timed such that the essential amounts of water are applied during the critical phase, but that moderate stress is allowed during the vegetative and maturity stages.^3^

Water is a critical component that limits growth, development, and output, particularly in semiarid and arid regions where plants are frequently exposed to drought stress, referred to as stages of water deficit stress.^4^ One of the main reasons crops fail globally is drought, which can lower average yields by 50% or more. Drought stress negatively affects a number of morpho-physiological attributes, which combined stunted plant development and productivity.^5^^,^^6^ It is well known that drought stress impairs a number of plant physiological and metabolic processes. It causes reduced growth, a decrease in water status and chlorophyll pigment, and changes in the properties of fluorescence.^7^

Drought stress increased the generation of reactive oxygen species (ROS) due to decreased light absorption and photosynthetic electron transport, resulting in photo oxidative destruction of photosystems.^8^ Oxidative damage to the cell membrane increases, whereas photosynthetic activity decreases. Plants have an antioxidant defense system, and increased production of antioxidants like superoxide dismutase (SOD), catalase (CAT), ascorbate peroxidase (ASP), and peroxidase (POD) helps to decrease reactive oxygen species (ROS) created during drought stress.^9^

During various phases of plant growth, the exogenous use of plant growth-regulators including ascorbic acid (AA) and salicylic acid (SA), can significantly enhance drought resistance.^10^^,^^11^ Ascorbic acid, also known as vitamin C, is an important antioxidant that regulates the generation of reactive oxygen species (ROS) during various biological activities in plants, including photosynthesis, respiration, proliferation, differentiation, and other metabolic processes.^12^ Ascorbic acid stimulates maize development under drought stress through various physiological and biochemical pathways.^13^ It functions as a strong antioxidant, scavenging reactive oxygen species (ROS) and reducing oxidative stress to protect cellular structures from injury. Ascorbic acid modulates stomatal motility, which improves water efficiency and reduces excessive water loss. It stabilizes chloroplast membranes and inhibits chlorophyll breakdown, ensuring long-term photosynthetic activity. It also enhanced the concentration of osmolytes, such as soluble carbohydrates and proline, which helps to maintain osmotic equilibrium and cellular turgor. Additionally, ascorbic acid affects enzymatic activity, improving the activity of antioxidant enzymes and thus lowering oxidative stress. Furthermore, it promotes root growth and ion homeostasis, resulting in a greater intake of nutrients and plant resilience. These combined effects help to promote biomass accumulation, resistance to stress, and total maize productivity under drought circumstances.^14^

In addition to being a natural growth hormone, salicylic acid (SA) also functions as a possible non-enzymatic antioxidant and regulates a wide range of biochemical processes occur in plants. It plays a vital role in alleviating drought stress in maize by improving antioxidant defense, regulating water balance, and enhancing nutrient intake.^15-17^ It activates stress-responsive pathways, boosting antioxidant enzymes that counteract the oxidative damage caused by reactive oxygen species (ROS). Salicylic acid decreases water loss by modifying stomatal behavior and increases water-use efficiency. It helps to retain chlorophyll content, which supports photosynthesis during droughts.^18^ Furthermore, salicylic acid enhances the accumulation of osmolytes, which helps with osmotic correction and cell turgor maintenance. It also promotes root growth and maintains ion homeostasis, resulting in efficient nutrient absorption. Salicylic acid enhances maize resilience through several processes, resulting in increased plant development even under drought conditions.^19^ It been noted that AA and SA lessen the consequences of water scarcity. The current study set out to assess how well SA and AA could counteract the detrimental effects of water scarcity and how they would affect the physiological and production characteristics of the maize crop.^20^

The use of salicylic acid and ascorbic acid has been observed to help alleviate drought stress. However, little research has been conducted to assess the combined effects of plant growth regulators on maize plants under drought stress, and more investigation is required. The study hypothesized that applying AA and SA to maize plants would ameliorate the negative effects of drought stress. The objectives of this study include (1) investigating the synergistic impact of ascorbic and salicylic acid use on drought stress tolerance in maize, with a focus on their impact on the antioxidant defense system and osmolyte accumulation, and (2) comparing the efficacy of ascorbic and salicylic acid, either separately and in combination, in lowering drought-induced oxidative stress and enhancing maize productivity during drought stress.

## Materials and methods

### Experimental setup, design, and treatments

A pot experiment was performed at the Wirehouse, Department of Agronomy, University of Agriculture, Faisalabad, to study the role of foliar-applied ascorbic acid and salicylic acid in maize to reduce the negative effects of drought stress. The treatments of the experiment includes: (1) drought stress (WW = well-watered at 85% FC, DS = drought stress at 40% FC), (2) foliar applications (NH = no hormone applied, DWS = distilled water spray, AA2 = ascorbic acid at 200 mg/L, SA2 = salicylic acid at 200 mg/L, and AASA = combined application of ascorbic and salicylic acid 100 mg/L + 100 mg/L) (Table 1). The field capacity (FC) was determined following the method of Pennypacker,^21^ while watering the plants to maintain a soil moisture 85 and 40% of its full capacity by checked daily weight of the pots. The maize variety (Pioneer-3875) was sown in pots (10 kg soil) having a size of 12 inches in length and 8 inches in diameter, and each pot contained 3 seeds. Drought stress and foliar applications were applied at the beginning of the tasseling stage (BBCH scale code 55) before silk emergence by limiting irrigation and simulating natural drought conditions.^22^ To make a foliar solution containing 200 mg ascorbic acid and 200 mg salicylic acid was dissolved in 1 L of distilled water. For the combined application, 100 mg of ascorbic acid was dissolved in 1 l of distilled water. Separately, 100 mg of salicylic acid was dissolved in a small amount of ethanol or NaOH (0.1 N), then mixed with the ascorbic acid solution and stirred well before being applied as a foliar spray. To ensure that the salicylic acid dissolves, add a small amount of ethanol. Thinning was done after 10 d of seed germination. The nutrient requirements were fulfilled with the Hoagland's nutrient solution.^23^ This experiment was arranged under a factorial arrangement in a completely randomized design (CRD) to apply the treatments. The experimental study consisted of a total of 30 pots, half of which were treated with drought conditions, with three replications each. The other half of the pots were treated with foliar ascorbic and salicylic acid applications, each with three replications.

**Table 1.** Experimental treatments.

Factors	Treatments
Drought stress (applied at inflorescence emergence on 50 DAS, BBCH scale code 50) (Weber and Bleiholder, 1990; Lancashire et al. 1991)	WW = well-watered at 85% FC DS = drought stress at 40% FC
Foliar applications (applied at inflorescence emergence on 50 DAS, BBCH scale code 50) (Weber and Bleiholder, 1990; Lancashire et al. 1991)	NH = no hormone applied DWS = distilled water spray AA2 = ascorbic acid at 200 mg/L SA2 = salicylic acid at 200 mg/L AA + SA = combined application of ascorbic acid and salicylic acid (100 mg/L + 100 mg/L)
Replications	3
The total pots (30) were divided into, Group I = well-watered pots (15 pots) Group II = drought stress (15 pots)	

Completely randomized design (CRD) was used to conduct the experiment.

### Morphological attributes

The plant growth was measured using metrics such as shoot and root length, fresh weight of the shoot and root, leaf area, number of leaves, and dried weight of shoot and root after harvesting. Weights were measured using an electronic balance, and lengths were measured with a meter rod.

### Photosynthetic pigments

Using the method described by Arnon,^24^ the amounts of carotenoids, total chlorophyll, and chlorophyll (a and b) were determined. The fresh leaf samples (0.1 g) were collected. The leaves were sliced, put in 5 ml of 80% acetone solution, and then left overnight at room temperature in the dark. The carotenoid contents were noted at an optical density of 480 nm with a spectrophotometer (IRMECO U2020, IRMECO Gmbh, Schwarzenbek, Germany). Using this same spectrophotometer, an optical density of the contents of both chlorophyll a and b were measured at 663 and 645 nm, respectively.

### Stress indicators

#### Malondialdehyde

A marker called malondialdehyde (MDA) is used to measure lipid peroxidation. The protocol used to measure MDA was the one described by Heath and Packer.^25^ The fresh leaf samples (0.2 g) were ground into 2 ml of 0.5% TCA, centrifuged the extract for 12 min at 12,000 rpm. After 30 min of heating in a water bath at 95 °C, a test tube was filled with 1 ml of supernatant and 4 ml of 0.5% TBA. The test tubes were then allowed to cool down on ice before being analyzed using a spectrophotometer at 532 and 600 nm.

#### Hydrogen peroxide

The content of hydrogen peroxide (H_2_O_2_) was evaluated following the procedure given by.^26^ The ice-chilled pestle and mortar were used to crush 0.2 g of fresh leafy material in 0.1% TCA (2 ml). The mixture was then centrifuged at 12,000 rpm for approximately 15 min. Added supernatant (0.5 ml), potassium phosphate buffer (0.5 ml), and KI (1 ml) into test tubes. The wavelength of the reaction mixture was measured with a spectrophotometer at 390 nm.

### Enzyme extraction

The freshly cut leaves, weighing 0.5 g, were ground up with five milliliters of 50 mM phosphate buffer solution (pH 7.8). The mixture was then centrifuged for 12 min at 12,000 rpm with the help of a centrifuge machine. The separated supernatant was collected and stored at −20 °C for further detection of antioxidant enzymes.

#### Determination of enzymatic antioxidants

The catalase activity was determined using the technique described by Chance and Maehly.^27^ A solution mixture was made by adding an enzyme extract (0.1 ml), phosphate buffer (50 mM), and H_2_O_2_ (5.9 mM). Then, using a spectrophotometer, noted the absorbance at 240 nm with 30-, 60-, and 90-s intervals. The protocol of Giannopolitis and Reis^28^ was followed to assess superoxide dismutase (SOD) activity. A reaction mixture was prepared by adding distilled H_2_O (400 μl), phosphate buffer (250 μl), L-methionine (100 μl), Triton X (100 μl), NBT (50 μl), enzyme extracts (50 μl), and riboflavin (50 μl). After 20 min of exposure to bright white light, the absorbance was measured at 560 nm, using a spectrophotometer. Peroxidase activity was measured using the Chance and Maehly^27^ approach. The enzymatic extract (0.1 ml), of potassium phosphate buffering (50 mM), guaiacol (20 mM), and H_2_O_2_ (40 mM). A spectrophotometer was utilized to check the absorbance of the mixture at 470 nm with 30-, 60-, and 90-s intervals.

### Determination of osmoprotectants

#### Proline

The proline content was determined by applying the procedure.^29^ After being ground to a fine consistency and combined with 5 ml of 10% sulfosalicylic acid, 0.25 g of fresh plant leaves were filtered through a Whatman filter paper. A test tube was filled with two milliliters of the filtrate, two milliliters of glacial acetic acid, and two milliliters of acid ninhydrin. The test tube was then submerged in a 75 °C water bath for one hour. The test tube was cooled to proceed the reaction. Added 4 ml of toluene in the test tubes, and it was vortexed for around 30 s.

#### Total soluble sugar

The total soluble sugar was estimated using the protocol of Handle.^30^ Test tubes containing a fresh leaf sample (0.1 g) and distilled water (5 ml) were vortexed for 30 s before being put in a water bath that was heated at 95 °C for about 20 min. After cooling the reaction mixture, the absorbance at 620 nm was measured using a spectrophotometer.

### Determination of inorganic ions

The procedure described by Allen et al.^31^ was used to determine the ionic contents of the maize plant. The digesting flask was taken, and 0.1 g of dried leaf biomass was added, submerged for three hours in HNO_3_ (5 ml). Three hours later, the digesting flasks were set on an electric heat plate at 350 °C. Then, 35% H_2_O_2_ was progressively added to produce a colorless, transparent solution. The mixture was filtered after being further diluted with distilled water to 25 ml. The ionic content of the filtrate samples (Na^+^, Ca^2+^, and K^+^) was determined using a flame photometer (Sherwood Famous Photometer-410, Sherwood Scientific Ltd., Cambridge, UK).

### Determining yield contributing-attributes

The yield attributes, such as cob length and diameter, were measured with the help of a measuring tape and a vernier caliper respectively. The number of grains was counted manually, while the biological yield and grain weight were recorded using an electronic weight balance.

### Statistical analysis

The experiment was carried out using a completely randomized design (CRD), with three replications to assure reliability. With the use of Statistics software, version 8.1, the recorded data was assessed at a significance level of 5%. Furthermore, the figures were created using Microsoft Excel (2016 edition) (Microsoft Corporation, Redmond, WA, USA). The origin (version 2021) and R-Studio software were used to create the correlation and heat map analysis.

## Results

### Foliar-applied ascorbic and salicylic acid improved the morphological attributes of maize under drought stress

The morphological indices of the maize plants were significantly affected under drought conditions and by foliar application of plant growth regulators. Drought stress was found to significantly reduce morphological attributes, including stem diameter (0.6%), the leaf area index (11%) and shoot fresh weight (12%), root fresh weight (12%), shoot dry weight (13%), and root dry weight (15%). On the other hand, foliar application of plants growth regulators helps to improve morphological characteristics by minimizing the detrimental effects of drought conditions on maize plants. However, under drought stress, foliar application of ascorbic acid as well as salicylic acid improved shoot length by 23% and 35%, and the leaf area index by 17% and 27%, respectively. When compared to the control condition, the combination foliar application of ascorbic acid and salicylic acid significantly increased shoot length by 44% and the leaf area index by 37% under drought stress (Table 2).

**Table 2.** Interactive effect of drought stress and ascorbic and salicylic acid application on morphological attributes of maize.

Drought stress	Treatments	Shoot fresh weight (g)	Root fresh weight (g)	Shoot dry weight (g)	Root dry weight (g)	Shoot length (cm)	Root length (cm)	Stem diameter (cm^2^)	Leaf area index (cm^2^)
WW	NH	13.1 ± 0.5efg	10.1 ± 0.3def	6.8 ± 0.2ef	6.3 ± 0.1de	60.5 ± 2.2def	35.3 ± 0.7efg	3.5 ± 0.01ef	4.6 ± 0.07fgh
	DWS	14.7 ± 0.4cdef	11.4 ± 0.4cde	7.7 ± 0.3cde	7.1 ± 0.1bcd	72.2 ± 2.3cd	38.5 ± 1cdef	3.6 ± 0.01cde	5.07 ± 0.1def
	AA2	16 ± 0.4bcd	12.4 ± 0.3bc	8.8 ± 0.2bc	7.8 ± 0.1bc	76 ± 2.1bc	42.3 ± 1.4bcd	3.6 ± 0.015bcd	5.49 ± 0.1bcd
	SA2	17.3 ± 0.3ab	13.5 ± 0.3ab	9.4 ± 0.2ab	8.2 ± 0.2ab	85 ± 2.5ab	45.5 ± 1.2ab	3.6 ± 0.01ab	5.94 ± 0.1ab
	AA + SA	19.3 ± 0.4a	14.4 ± 0.5a	10.3 ± 0.2a	8.9 ± 0.4a	94.2 ± 2.7a	49.1 ± 1.6a	3.7 ± 0.005a	6.39 ± 0.1a
DS	NH	11.5 ± 0.4g	8.8 ± 0.2f	5.7 ± 0.1f	5.2 ± 0.08f	52.5 ± 2.4f	31 ± 1.1g	3.5 ± 0.005f	4.11 ± 0.1h
	DWS	12.7 ± 0.6fg	9.8 ± 0.2ef	6.6 ± 0.2ef	5.9 ± 0.2ef	59.5 ± 1.9ef	34.2 ± 1.1fg	3.5 ± 0.01ef	4.48 ± 0.09gh
	AA2	14.1 ± 0.6def	11 ± 0.4cde	7.4 ± 0.2de	6.5 ± 0.1de	64.8 ± 2.4cde	37.6 ± 0.8def	3.6 ± 0.01de	4.82 ± 0.1efg
	SA2	15.2 ± 0.3bcde	12 ± 0.4bcd	8.3 ± 0.1bcd	7 ± 0.1cd	71 ± 2.3cde	40.6 ± 1.2bcde	3.6 ± 0.01cde	5.24 ± 0.1cde
	AA + SA	16.6 ± 0.5bc	12.7 ± 0.3abc	9.3 ± 0.2ab	7.6 ± 0.2bc	75.9 ± 2.4bc	44.3 ± 1.8abc	3.6 ± 0.1bc	5.67 ± 0.1bc

Values represent means ± standard error of three replicates. Same letter sharing by means for a parameter indicates that they do not vary significantly based on the Tuckey test α = 0.05. Drought (WW = well-watered at 85% FC, DS = drought stress at 40% FC), hormonal applications (NH = no hormone applied, DWS = distilled water spray, AA2 = ascorbic acid at 200 mg/L, SA2 = salicylic acid at 200 mg/L, and AA + SA = combined application of ascorbic acid and salicylic acid 100 mg/L + 100 mg/L)

### Foliar-applied ascorbic and salicylic acid improved the photosynthetic pigments of maize under drought stress

**Figure 1.**
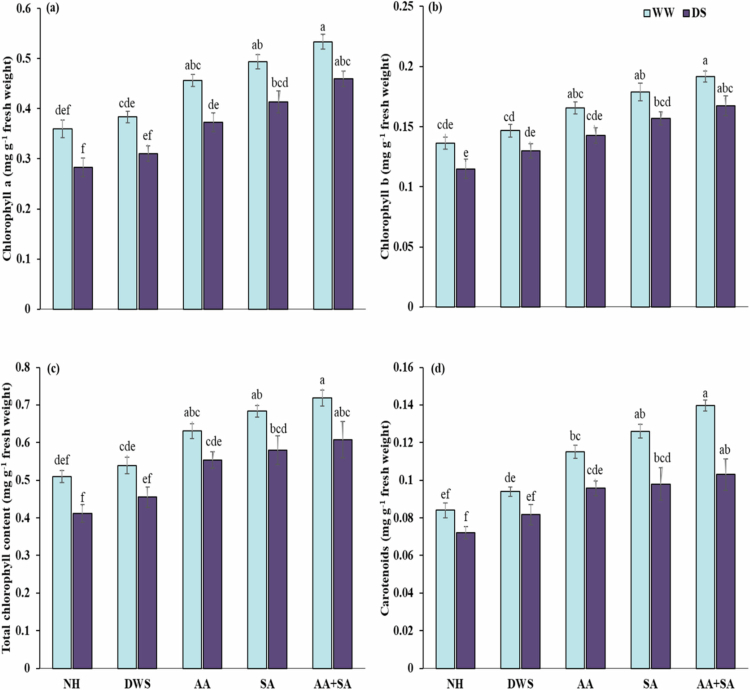
Effects of foliar-applied ascorbic and salicylic acid on (a) chlorophyll a, (b) chlorophyll b, (c) total chlorophyll content, and (d) carotenoid content of maize plants under normal and drought stress conditions. Various letters above the mean (three replicates) indicate a significant difference between the means at *p* < 0.05 according to the Tukey HSD test. Error bars represent the standard errors.

In maize plants, drought stress and foliar application of growth regulators showed a significant impact on the photosynthetic pigment. Drought stress was observed to considerably diminish photosynthetic pigments, including total chlorophyll (15%) and carotenoids (19%) compared with the control. In contrast, foliar application of growth regulators significantly improved photosynthetic pigments by mitigating the negative effects of drought stress in maize plants. However, foliar use of ascorbic acid and salicylic acid enhanced total chlorophyll by 34% and 40%, respectively, and carotenoids by 32% and 36%, respectively, under drought stress conditions. In comparison to the control condition, the combination of foliar spray of ascorbic acid and salicylic acid significantly increased the total chlorophyll by 47% and carotenoids by 43% under drought stress (Figure 1).

### Foliar-applied ascorbic and salicylic acid altered stress indicators production under drought stress

**Figure 2.**
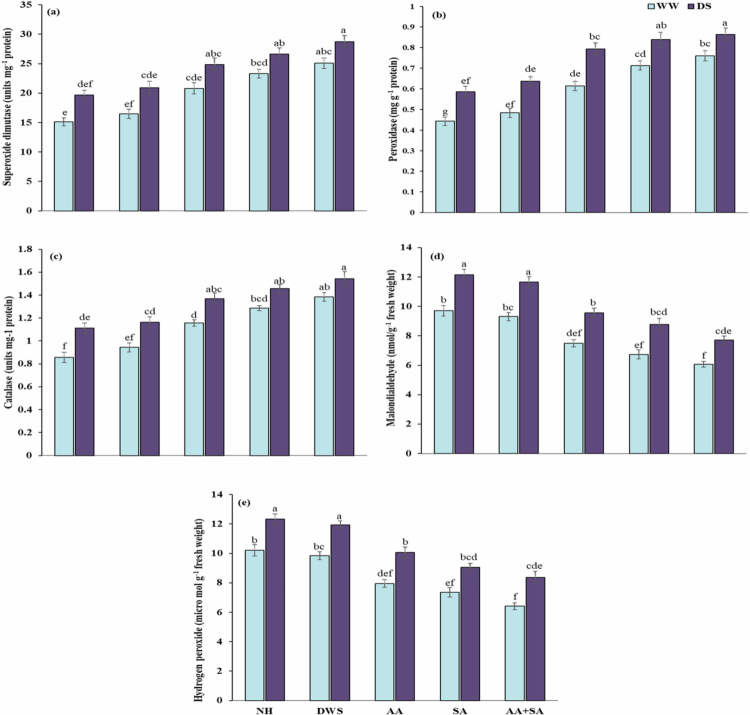
Effects of foliar-applied ascorbic and salicylic acid on (a) SOD, (b) POD, (c) CAT, (d) MDA, and (e) H_2_O_2_ content of maize plants under normal and drought stress conditions. According to the Tukey HSD test, various letters above the mean (three replicates) indicate a significant difference between the means at *p* < 0.05. Error bars represent the standard errors.

Stress indicators were significantly affected under drought and foliar application of growth regulators. Drought stress was found to increase the production of stress indicators such as MDA and H_2_O_2_ by 26% and 23%, respectively as compared to control conditions. In contrast, the foliar applied growth regulators mitigate the negative effects of overproduced stress indicators. However, under drought stress circumstances, foliar treatment of ascorbic acid and salicylic acid reduces the MDA concentration by 21% and 27%, respectively, and H_2_O_2_ by 18% and 26%, respectively. Among the applications, the combined foliar application of ascorbic acid and salicylic acid reduced the MDA concentration by 36% and H_2_O_2_ by 32% under drought stress when compared to the control (well-watered) condition (Figure 2).

### Foliar-applied ascorbic and salicylic acid improved enzymatic antioxidant activity under drought stress

Enzymatic antioxidants showed significant variation under drought and foliar-applied plant growth regulators in maize plants. It was found that, in comparison to the control group, drought stress significantly raised antioxidant levels of SOD by 19%, POD by 23%, and CAT by 18%. The enhanced enzymatic antioxidants were insufficient to cope with the negative effects of drought stress. In contrast, foliar-applied plant growth regulators can considerably increase these enzymatic antioxidants' activity in dealing with the adverse effects of drought conditions on maize plants. However, under drought stress circumstances, foliar treatment of ascorbic acid and salicylic acid raised SOD by 26% and 35%, and POD by 35% and 46%, respectively. Among the applications, the combined foliar application of ascorbic acid and salicylic acid showed a greater increase in SOD (45%) and POD (47%) under drought stress in comparison with the control (Figure 2).

### Foliar-applied ascorbic and salicylic acid increased osmoprotectants accumulation under drought stress

**Figure 3.**
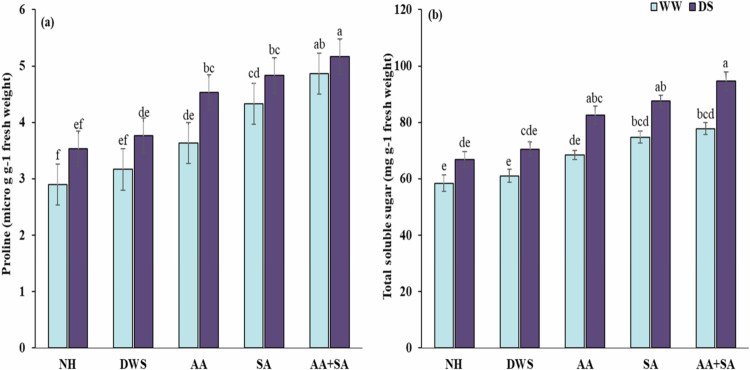
Effects of foliar-applied ascorbic and salicylic acid on the (a) proline and (b) total soluble sugar content of maize plants under normal and drought stress conditions. According to the Tukey HSD test, various letters above the mean (three replicates) indicate a significant difference between the means at *p* < 0.05. The error bars represent the standard errors.

Osmoprotectants in maize plants were significantly impacted by drought stress and the foliar application of plant growth regulators. Drought stress significantly increased osmoprotectants such as proline and total soluble sugars (TSS) by 15% and 19%, respectively, as compared to the control. Plant growth regulators, on the other hand, can significantly increase proline and total soluble sugar levels by mitigating the negative effects of drought stress on the growth of maize. However, under drought stress, foliar application of ascorbic acid and salicylic acid raised proline by 28% and 36%, and TSS by 9% and 14%, respectively. Among the applications, the combined application of ascorbic acid and salicylic acid showed a greater increase in proline by 46% and TSS by 20% under drought stress compared to the control conditions (Figure 3).

### Foliar-applied ascorbic and salicylic acid altered nutrient uptake under drought stress

Nutrient uptake of the maize plant showed significant variation under the influence of drought stress and foliar-applied growth regulators. Drought stress was found to increase the Na^+^ by 11% while decreasing K^+^ by 13%, and Ca^2+^ by 11% as comparison to the control. On the other hand, foliar-applied growth regulators play a positive role in ionic homeostasis under drought stress. However, under drought stress conditions, foliar treatment with ascorbic acid and salicylic acid lowered Na^+^ by 13% and 18%, respectively, while increasing the uptake of K^+^ by 17% and 26%, and Ca^2+^ by 13% and 20%, respectively. Among the applications, the combined application of ascorbic acid + salicylic acid resulted drop in Na^+^ by 26%, while increasing the uptake of K^+^ by 33%, and Ca^2+^ by 22% under drought conditions as compared to the control condition (Table 3).

**Table 3.** Interactive effect of drought stress and ascorbic and salicylic acid application on ionic content of maize.

Drought stress	Treatments	Na^+^ in leaves mg/g DW	K^+^ in leaves mg/g DW	Ca^2+^ in leaves mg/g DW
WW	NH	7.3 ± 0.2abc	3.8 ± 0.1a	1.7 ± 0.07a
	DWS	6.8 ± 0.2abcd	3.4 ± 0.08ab	1.6 ± 0.03ab
	AA2	6.4 ± 0.1bcde	3.2 ± 0.08abc	1.5 ± 0.07abc
	SA2	5.7 ± 0.2de	2.9 ± 0.1bcd	1.4 ± 0.05bcd
	AA + SA	5.1 ± 0.2e	2.5 ± 0.08de	1.3 ± 0.04cd
DS	NH	8 ± 0.2a	3.3 ± 0.08abc	1.6 ± 0.05abc
	DWS	7.6 ± 0.3ab	3 ± 0.08bcd	1.4 ± 0.04bcd
	AA2	7 ± 0.2abcd	2.7 ± 0.08cde	1.3 ± 0.03cd
	SA2	6.5 ± 0.3bcd	2.4 ± 0.1de	1.2 ± 0.05d
	AA + SA	5.9 ± 0.2cde	2.2 ± 0.08e	1.2 ± 0.04d

Values represent means ± standard error of three replicates. Same letter sharing by means for a parameter indicates that they do not vary significantly based on the Tuckey test α = 0.05. Drought (WW = well-watered at 85% FC, DS = drought stress at 40% FC), hormonal applications (NH = no hormone applied, DWS = distilled water spray, AA2 = ascorbic acid at 200 mg/L, SA2 = salicylic acid at 200 mg/L, and AA + SA = combined application of ascorbic acid and salicylic acid 100 mg/L + 100 mg/L).

### Foliar-applied ascorbic and salicylic acid increased yield-contributing attributes under drought stress

Yield-related indices are significantly influenced by the foliar application of growth regulators and drought stress conditions. Drought stress was found to significantly decrease yield attributes such as the cob length (by 19%), the number of rows per cob (by 13%), the number of grains per row (by 19%), the 100-grain weight (by 26%), and the biological yield by 14%, as compared to the control. In contrast, foliar application of plant growth regulators plays a positive role in improving yield indices under drought stress. However, foliar application of ascorbic acid and salicylic acid increased the cob length by 32% and 42% and the 100-grain weight by 22% and 34%, respectively, under drought stress conditions. Among the applications, the combined foliar application of ascorbic acid + salicylic acid showed a significant increase in cob length by 48% and 100-grain weight by 49% under drought stress as compared to the control condition (Table 4).

**Table 4.** Interactive effect of drought stress and ascorbic and salicylic acid application on growth and yield attributes of maize.

Drought stress	Treatments	Cob length (cm)	Cob diameter (cm)	Number of grains per cob	100-grain weight (g)	Biological yield (t ha^−1^)	Grain yield (t ha^−1^)	Harvest index (%)
WW	NH	10.7 ± 0.3de	2.3 ± 0.02de	229 ± 4.8fg	21.1 ± 0.8bcde	16.67 ± 0.6de	4.9 ± 0.1ef	28.5 ± 1.3a
	DWS	12.1 ± 0.2cd	2.6 ± 0.01bcd	253 ± 5.1de	22.5 ± 1bc	18.4 ± 0.6cde	5.2 ± 0.1def	27.6 ± 0.9ab
	AA2	13.3 ± 0.3bc	2.9 ± 0.01abc	286 ± 3.9c	24 ± 1.2ab	20.7 ± 0.6bc	5.5 ± 0.1bcd	26.9 ± 1.1ab
	SA2	14.9 ± 0.5ab	3.1 ± 0.01ab	316 ± 4.6ab	25.6 ± 0.8ab	23 ± 0.8ab	6 ± 0.1ab	26.1 ± 1.2abc
	AA + SA	16.1 ± 0.3a	3.2 ± 0.008a	336 ± 4.5a	27.3 ± 0.9a	25.8 ± 1a	6.2 ± 0.1a	24.2 ± 0.4abc
DS	NH	8.5 ± 0.4f	2.1 ± 0.006e	183 ± 3.3h	14.3 ± 0.7f	12.8 ± 0.5f	3.5 ± 0.1g	25.9 ± 1.4abc
	DWS	9.8 ± 0.4ef	2.3 ± 0.008de	207 ± 4g	16.4 ± 0.9ef	15.3 ± 0.6ef	3.7 ± 0.1fg	24.5 ± 0.5abc
	AA2	11.3 ± 0.4de	2.5 ± 0.008cde	240 ± 3.2ef	17.6 ± 0.6def	17 ± 0.4de	4 ± 0.1ef	23.6 ± 0.4bc
	SA2	12.1 ± 0.3cd	2.7 ± 0.01bcd	262 ± 5.2d	19.2 ± 0.8cde	19.2 ± 0.6cd	4.4 ± 0.1cde	23 ± 0.9b c
	AA + SA	12.6 ± 0.5cd	2.9 ± 0.008abc	301 ± 5.3bc	21.4 ± 1.2bcd	22.4 ± 0.4bc	4.7 ± 0.1bc	21.4 ± 0.4c

Values represent means ± standard error of three replicates. Same letter sharing by means for a parameter indicates that they do not vary significantly based on the Tuckey test α = 0.05. Drought (WW = well-watered at 85% FC, DS = drought stress at 40% FC), hormonal applications (NH = no hormone applied, DWS = distilled water spray, AA2 = ascorbic acid at 200 mg/L, SA2 = salicylic acid at 200 mg/L, and AA + SA = combined application of ascorbic acid and salicylic acid at 100 mg/L + 100 mg/L).

### Heat map and correlation analysis

**Figure 4.**
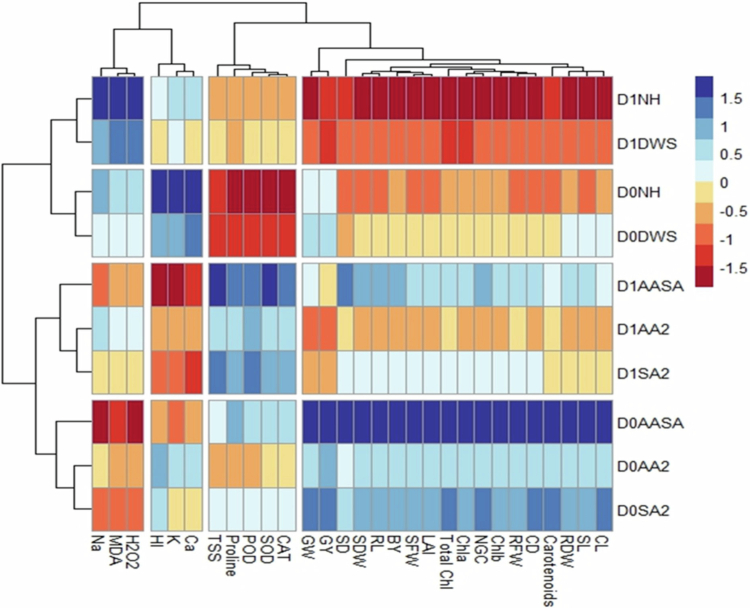
Heat map analysis across the various recorded attributes of maize plant under the interactive effects of drought stress and ascorbic and salicylic acid foliar application. The abbreviations of the indices are SFW = shoot fresh weight, RFW = root fresh weight, SDW = shoot dry weight, RDW = root dry weight, SL = shoot length, RL = root length, SD = stem diameter, LAI = leaf area index, Chl a = chlorophyll a, Chl b = chlorophyll b, Total Chl = total chlorophyll contents, SOD = superoxide dismutase, POD = peroxide dismutase, CAT = catalase, H_2_O_2_ = hydrogen peroxide, MDA = malondialdehyde, proline, TSS = total soluble sugar, Na = sodium in leaves, K = potassium in leaves, Ca = calcium in leaves, CL = Cob length, CD = Cob diameter, NGC = number of grains per cob, GW = grain weight, BY = biological yield, GY = grain yield, and HI = harvest index. The treatments group include D0 = control (well-watered), D1 = drought stress, NH = no hormone applied, DWS = distilled water spray, AA2 = ascorbic acid at 200 mg/L, SA2 = salicylic acid at 200 mg/L, and AASA = AA2 + SA2 (100 mg/L + 100 mg/L).

The analysis of heat map was done among the recorded morphological attributes, photosynthetic pigments, biochemical indices, ionic contents in leaves, and yield-contributing attributes of the maize plant under the influence of drought stress and single and combined foliar-applied ascorbic and salicylic acid. The variations of colors in the boxes of the heat map were associated with the scale color from blue (strong positive) to red (strong negative). It was observed that a significant improvement in the morphological indices, photosynthetic pigments, and yield attributes, while stress indicators and sodium ions in the leaves under the treatment D0AASA. On the other hand, the above-mentioned recorded attributes showed a decrease under D1NH treatment, except for stress indicators and sodium ions, which were increased under this treatment. Increased enzymatic antioxidant activity and accumulation of osmolytes were observed under D1AASA treatment, enabling the plant to mitigate the adverse effects of drought stress (Figure 4).

**Figure 5.**
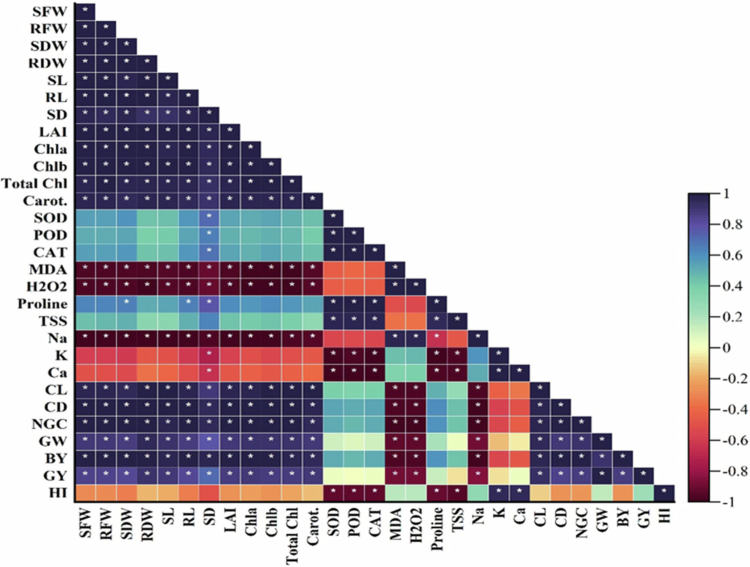
Correlation analysis was done among the various recorded attributes of the maize plant under the interactive effects of drought stress and ascorbic and salicylic acid foliar application. The abbreviations of the indices are listed in the caption of Figure 4.

Correlation analysis was created between various morphological attributes, photosynthetic pigments, biochemical indices, ionic contents in leaves, and yield-contributing attributes of the maize plant under the interactive effects of drought stress and single and combined foliar-applied ascorbic and salicylic acid. A strong positive correlation was found in the morphological indices, and photosynthetic pigments with the yield-contributing indices. The enzymatic antioxidants and osmolytes was found a slight positive correlation with these attributes. In contrast, stress indicators (MDA and H_2_O_2_) and sodium ions showed a strong negative correlation with the maize plant's above-mentioned indices (Figure 5).

## Discussion

Abiotic unfavorable circumstances have a considerable impact on agricultural productivity and global food production, and climate change contributes to these challenges. Drought, caused by changing rainfall patterns, increasing atmospheric carbon dioxide, and rising temperatures, is a major abiotic stress that impedes agricultural productivity.^32^^,^^33^ Drought-stressed maize plants are adversely affected by oxidative damage, which contributes to reducing the efficiency of physiological mechanisms through destroying photosynthetic pigments, ultimately cause sever reduction of the growth and production (Tables 2 and 4). The application of plant growth promoters such as salicylic and ascorbic acid take positive part to boost the plant tolerance mechanisms against drought stress.^34^^,^^35^ The findings of the present study demonstrate that foliar application of the above-mentioned growth regulators significantly reduces the harmful effects of drought-induced oxidative damage through improving the enzymatic antioxidant activity and accumulation of secondary metabolites, which leads to enhanced growth and productivity of the maize plant (Tables 2–4 and Figures 1–3).

The drought-exposed maize plant showed a reduction in morphological attributes, while foliar-applied growth regulators significantly improved under drought as well as control conditions. According to a prior study,^36^ the morphological parameters significantly decrease under drought stress. The decrease in cell proliferation and hindering of cell elongation caused by an interruption in the water transport from the xylem to the adjoining elongating cells, transpiration loss due to the closure of stomatal pores, and decreased water potential of the soil are the causes of this reduction.^37^ In contrast to control conditions, foliar applications of plant growth regulators, such as salicylic and ascorbic acid, both separately and in combination, significantly improve morphological parameters when drought stress occurs.^38^ This increase is caused by salicylic acid and ascorbic acid, which also function as signaling molecules to activate stress-responsive genes and pathways while boosting antioxidant enzyme activity and controlling osmotic equilibrium, which may provide tolerance contrary to water injuries by maintaining a high tissue water potential.^39^

The photosynthetic attributes showed a significant decrease under drought stress as compared to control conditions (Table), as reported previous study.^40^ Stomata stay tight due to water stress, which hinders CO_2_ fixation and its conversion into a complex, energy-rich molecule, hence delaying photosynthesis, and the over-generation of ROS causes the degradation of photosynthetic pigments.^40^^,^^41^ However, individual and combined foliar applications of plant growth regulators such as ascorbic and salicylic acid show a significant rise in the photosynthetic pigments under drought stress as compared to the control conditions.^42^ Ascorbic and salicylic acid enhance physiological activities during drought stress, which protect the chlorophyll contents from oxidative damage, and contribute to a rise in the content of maize photosynthetic pigments.^43-45^

Stress indicators, such as increased ROS over-generation during water shortages, cause plants to engage in a variety of adaptation strategies, such as tolerance, escape, and evasion. Producing antioxidant enzymes is the most efficient reaction among these; they scavenge excess ROS by activating powerful antioxidant defenses. The previous studies reported that drought-exposed maize plants exhibited increased antioxidant activity compared to control circumstances.^46^ This rise results from the overproduction of ROS linked to lipid peroxidation and MDA production, which are indicators of oxidative stress brought on by abiotic stressors.^47^ However, when compared to control circumstances, the production of antioxidant enzymes under drought stress is significantly increased by both solo and combined foliar treatments of salicylic acid and ascorbic acid.^48^ This rise results from AsA scavenging ROS (H_2_O_2_) and MDA, which is linked to stress tolerance. Additionally, it improves oxidative defense, which supports plant growth and development under stressful environments.^49^

The overabundance of H_2_O_2_ and MDA in maize demonstrated that drought stress dramatically elevated lipid peroxidation, according to the current study's findings. Plants under drought stress experience oxidative damage as a result of increased lipid peroxidation levels and ROS overproduction, which negatively impacts the metabolic machinery and morphological characteristics of the plants.^50^^,^^51^ However, the detrimental effects of drought stress on maize were significantly overcome by individual and combined foliar treatments of salicylic acid and ascorbic acid. The previous investigations reported that foliar-applied ascorbic and salicylic acid reduced the H_2_O_2_ and MDA in maize under drought stress.^52^ By decreasing MDA accumulation and scavenging H_2_O_2_, the increase of antioxidant and osmolyte activity may aid in detoxifying the harmful effects of drought.^53^

According to a prior study, total soluble sugars (TSS) and proline levels rise in water-deficient plants in comparison to control conditions.^54^ The increased antioxidant defense system, osmoprotective mechanism, decreased oxidative stress, and improved synthesis of appropriate solutes are causes of this rise in proline content and TSS.^55^^,^^56^ However, proline and total soluble sugar synthesis significantly increase when ascorbic acid and salicylic acid are applied exogenously, both individually and in combination.^57^ This rise in proline content is linked to improved plant tolerance because it helps with cellular osmotic adjustment, ROS detoxification, protecting membrane integrity, nutritional homeostasis, ion compartmentalization, and protecting plant organelles and cells from ROS. It also affects a number of enzymes that activate various physiological and signaling pathways.^58^^,^^59^

The earlier research has demonstrated that in maize plants exposed to drought stress decreased potassium content, while sodium content increased in comparison to the control condition.^60^ This creates a negative link between sodium elements and other nutrients, as well as an ionic imbalance brought on by the cells' plasma membrane disorder in integrity.^61^ However, foliar-applied both ascorbic and salicylic acid lead to the regulation of nutrient uptake and ionic balance in plants, including boosting nutrient uptake and decreasing sodium (Na^+^) levels.^62^

The present study reported a dramatic decrease in calcium contents (Ca^2+^) under salt stress in maize leaves. Higher Na^+^ levels in the soil change the osmotic pressure in plant cells, which prevents the plants from absorbing Ca^2+^, other essential nutrients, and water.^63^ Nonetheless, under drought stress, ascorbic and salicylic acid foliar applications, both separately and in combination, significantly increased Ca^2+^ uptake while reducing Na^+^ concentrations.^64^ Because of the cell's homeostasis and the stability of Ca^2+^ ions under stress, this improvement occurs. As a result, Ca^2+^ is an essential metabolic regulator that promotes the removal of Na^+^.^65^

Water stress during the reproductive stage was found to reduce yield indices in the current study. This reduction was more pronounced in pots that experienced stress following the tasseling stage, as previously documented in studies.^66^ Decreased photosynthesis, increased leaf senescence, and sink constraints, reduced pollen production and fertility, along with a drop in grain quantity, are the causes of this loss in yield attributes.^67^ Nonetheless, ascorbic and salicylic acid, two plant growth regulators, when applied topically, increase the yield parameters.^68^ This rise is the result of assimilated transfer to the grains and increased photosynthesis, greater cell division and development, which raise fertility and pollen production.^69^ Because they are involved in primary physiological, metabolic, and physical processes, AA and SA are essential for controlling plant development. Internal processes like growth and development significantly alter the levels of AA and SA in plants.

## Conclusion

The current study indicated significant effects of AA and SA on various maize parameters. The results suggest that a combined foliar-applied AA and SA at the beginning of maize's reproductive stage is highly effective for improving all growth and yield indices, especially at the onset of the tasseling stage. Plant growth, SPAD-chlorophyll values, physiological, and yield attributes were impacted under drought stress. However, AA and SA foliar application play a positive role in mitigating stress by activating the plant defense system. The combined foliar application of AA and SA (100 mg/L + 100 mg/L) is therefore advantageous under water stress, as it significantly reduces the adverse effects of drought on maize and promotes growth and yield during critical stages of plant development. Future research should examine the root absorption mechanism of AA, its biochemical and metabolic effects, and its interactions with other antioxidants under various stress conditions. Understanding how AA is transmitted over long distances could enhance plant stress resilience. Additionally, more research on SA treatment during the tasseling stage is necessary to optimize drought resistance in maize and boost agricultural productivity.

## Data Availability

All data generated or analyzed during this study are included in this published article.
